# Palmitoylethanolamide Inhibits Glutamate Release in Rat Cerebrocortical Nerve Terminals

**DOI:** 10.3390/ijms16035555

**Published:** 2015-03-11

**Authors:** Tzu-Yu Lin, Cheng-Wei Lu, Chia-Chan Wu, Shu-Kuei Huang, Su-Jane Wang

**Affiliations:** 1 Department of Anesthesiology, Far-Eastern Memorial Hospital, Pan-Chiao District, New Taipei City 22060, Taiwan; E-Mails: drlin1971@gmail.com (T.-Y.L.); drluchengwei@gmail.com (C.-W.L.); chiachanc@yahoo.com.tw (C.-C.W.); nskh9450n@yahoo.com.tw (S.-K.H.); 2 Graduate Institute of Basic Medicine, Fu Jen Catholic University, No. 510, Zhongzheng Rd., Xinzhuang Distinct, New Taipei City 24205, Taiwan; 3 School of Medicine, Fu Jen Catholic University, No. 510, Zhongzheng Rd., Xinzhuang Distinct, New Taipei City 24205, Taiwan; 4 Department of Mechanical Engineering, Yuan Ze University, Taoyuan 320, Taiwan

**Keywords:** PEA, glutamate release, cerebrocortical nerve terminals, voltage-dependent Ca^2+^ channels, cannabinoid CB1 receptors, protein kinase A

## Abstract

The effect of palmitoylethanolamide (PEA), an endogenous fatty acid amide displaying neuroprotective actions, on glutamate release from rat cerebrocortical nerve terminals (synaptosomes) was investigated. PEA inhibited the Ca^2+^-dependent release of glutamate, which was triggered by exposing synaptosomes to the potassium channel blocker 4-aminopyridine. This release inhibition was concentration dependent, associated with a reduction in cytosolic Ca^2+^ concentration, and not due to a change in synaptosomal membrane potential. The glutamate release-inhibiting effect of PEA was prevented by the Cav2.1 (P/Q-type) channel blocker ω-agatoxin IVA or the protein kinase A inhibitor H89, not affected by the intracellular Ca^2+^ release inhibitors dantrolene and CGP37157, and partially antagonized by the cannabinoid CB1 receptor antagonist AM281. Based on these results, we suggest that PEA exerts its presynaptic inhibition, likely through a reduction in the Ca^2+^ influx mediated by Cav2.1 (P/Q-type) channels, thereby inhibiting the release of glutamate from rat cortical nerve terminals. This release inhibition might be linked to the activation of presynaptic cannabinoid CB1 receptors and the suppression of the protein kinase A pathway.

## 1. Introduction

Palmitoylethanolamide (PEA) is an endogenous lipid belonging to the family of fatty acid ethanolamides [1]. PEA has received considerable attention because of its low toxicity and many pharmacological activities such as anti-inflammatory, analgesic, and immunomodulatory effects [2,3,4]. PEA is abundant in the central nervous system (CNS) and exerts neuroprotective effects [2,3,4]. *In vitro* studies have demonstrated, for example, that PEA protects against oxidative stress or neurotoxin-induced neuronal death in cultured hippocampal cells [5,6,7]. Moreover, PEA administration has been reported to reduce brain damage and improve behavioral dysfunctions in several experimental models of CNS injury and disease, including epilepsy, cerebral ischemia, stroke, Alzheimer’s disease, and Parkinson’s disease [8,9,10,11,12,13,14]. These findings suggest that PEA acts as an endogenous protective factor of the brain; however, the precise mechanisms involved in this role are unclear.

In the CNS, glutamate functions as a major excitatory neurotransmitter to regulate normal neurotransmission and synaptic plasticity [15,16]. However, excessive glutamate release following the overactivation of glutamate receptors can induce neuronal death, a phenomenon known as excitotoxicity. This process has been implicated in the pathogenesis of numerous brain diseases including traumatic brain injury, stroke, epilepsy, Alzheimer’s disease, Parkinson’s disease, and others [17,18,19]. The blockade of glutamate neurotransmission, such as by glutamate receptor antagonists, has conferred neuroprotection in several *in vitro* and *in vivo* studies [20,21]; however, the occurrence of numerous side effects such as ataxia, psychotic effects, and memory impairment makes it unsuccessful in the clinic [22,23]. Therefore, a reduction in glutamate release may be a more promising neuroprotective strategy than a direct glutamate receptor blockade.

Although PEA is present in the brain and exerts a neuroprotective-like effect, no data are available on the effect of PEA on glutamate release. Therefore, the present work assessed the effects and possible mechanism of PEA on glutamate release from rat cerebrocortical nerve terminals (synaptosomes), a preparation by which presynaptic effects could be directly investigated, excluding extrasynaptic and polysynaptic events and the non-neuronal release of glutamate [24]. Using an established method for examining endogenous glutamate release [25], we found that PEA greatly inhibited glutamate release from synaptosomes by suppressing Ca_v_2.1 (P/Q-type) channels and protein kinase A activity. Furthermore, this release inhibition likely depended, at least in part, on the activation of presynaptic cannabinoid CB1 receptors.

## 2. Results

### 2.1. Effect of Palmitoylethanolamide (PEA) on the Release of Glutamate Evoked by 4-Aminopyridine in Rat Cerebrocortical Synaptosomes

**Figure 1:**
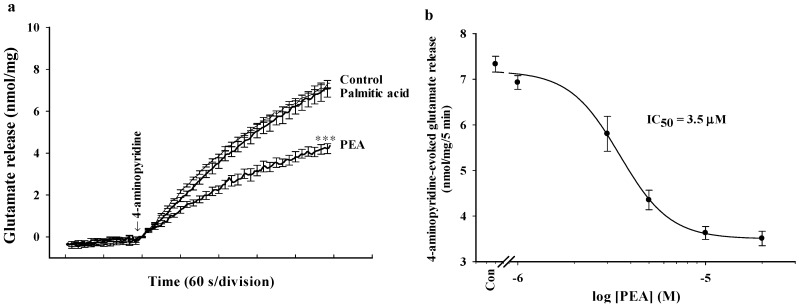
Palmitoylethanolamide (PEA) inhibits 4-aminopyridine-evoked release of glutamate in rat cerebrocortical nerve terminals. (**a**) Glutamate release was evoked by the addition of 1 mM 4-aminopyridine in the absence (control) and in the presence of PEA (5 μM) or palmitic acid (10 μM), added 10 min prior to the addition of 4-aminopyridine; (**b**) Concentration-effect relationship of PEA (1–20 μM) on 4-aminopyridine-induced glutamate release. Results are mean ± SEM of 5–14 independent experiments. *** *p* < 0.001 *versus* control group.

Synaptosomes were purified from the cerebral cortex of rats and exposed to 4-aminopyridine, a potassium channel blocker that opens voltage-dependent Ca^2+^ channels and induces the release of glutamate [26]. As shown in Figure 1a, under synaptosomes incubated in the presence of 1.2 mM CaCl_2_, the release of glutamate evoked by 1 mM 4-aminopyridine was 7.3 ± 0.2 nmol/mg/5 min. Preincubation of synaptosomes with 5 μM PEA for 10 min reduced the release of glutamate evoked by 4-aminopyridine to 4.2 ± 0.2 nmol/mg/5 min (*t*(13) = 9.67; *p* < 0.001; Figure 1a). The IC_50_ value for the PEA-mediated inhibition of 4-aminopyridine-evoked glutamate release, derived from a dose-response curve, was 3.5 μM (Figure 1b). Basal glutamate release was not altered by PEA. In addition, the specificity of the effect of PEA was evaluated using palmitic acid. Palmitic acid (10 µM) had no effect on the 4-aminopyridine (1 mM)-evoked release of glutamate (*t*(8) = 0.03; *p* = 0.98; Figure 1a).

### 2.2. Effect of Calcium Chelation, dl-Threo-β-benzyloxyaspartate (dl-TBOA), and Bafilomycin A1 on the Inhibition of 4-Aminopyridine-Evoked Glutamate Release by PEA

**Figure 2:**
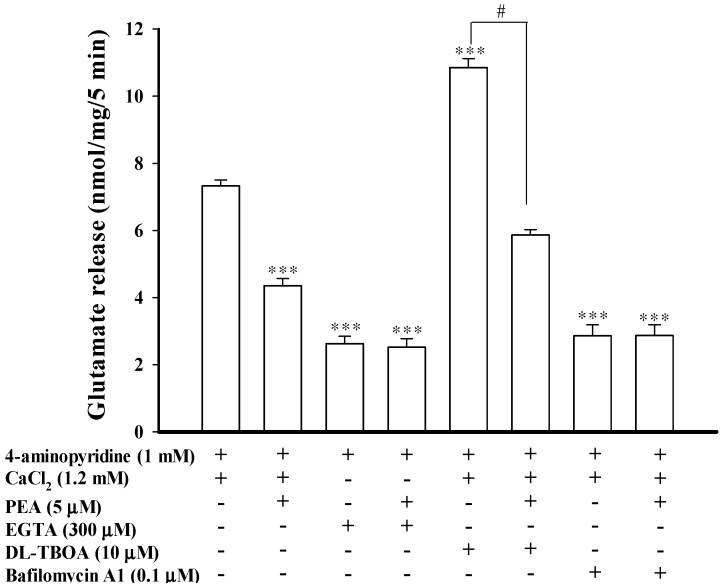
PEA-mediated inhibition of 4-aminopyridine-evoked glutamate release is due to a decrease in vesicular exocytosis. Bar graph showing glutamate release evoked by 1 mM 4-aminopyridine in the absence (control) or presence of 5 μM PEA, 300 μM EGTA (without CaCl_2_), 300 μM EGTA (without CaCl_2_) and 5 μM PEA; 10 μM dl-TBOA, 10 μM dl-TBOA and 5 μM PEA, 10 μM dl-TBOA and 5 μM PEA; 0.1 μM bafilomycin A1; or 0.1 μM bafilomycin A1 and 5 μM PEA. EGTA, dl-TBOA, or bafilomycin A1 were added 20 min before depolarization, while PEA was added 10 min before depolarization. Results are mean ± SEM of 5 independent experiments. *** *p* < 0.001 *versus* control group. ^# ^*p* < 0.05 *versus* the dl-TBOA-treated group.

The 4-aminopyridine-evoked release of glutamate from synaptosomes is known to have two components: the Ca^2+^-dependent fraction, which relies on synaptic vesicle fusion with the plasma membrane, and the Ca^2+^-independent fraction, which is attributed to the reversal of the glutamate transporter [26,27]. Thus, we examined the effect of PEA on the Ca^2+^-independent component of 4-aminopyridine-evoked glutamate release that can be estimated in an extracellular Ca^2+^-free solution containing 300 μM EGTA. Figure 2 shows that the Ca^2+^-independent release of glutamate evoked by 1 mM 4-aminopyridine was 2.7 ± 0.3 nmol/mg/5 min (*t*(9) = 14.16; *p* < 0.001), and this release was not affected by PEA at 5 μM (2.5 ± 0.2 nmol/mg/5 min; *p* = 0.95). In addition, dl-threo-β-benzyl-oxyaspartate (dl-TBOA), a glutamate reuptake inhibitor, or bafilomycin A1 (0.1 μM), a vesicular transporter inhibitor, was used to examine the effect of PEA. dl-TBOA (10 μM) did not affect basal glutamate release (0.03 ± 0.03 nmol/mg/5 min), but it increased the 4-aminopyridine-evoked glutamate release (7.2 ± 0.2 nmol/mg/5 min) to 10.8 ± 0.3 nmol/mg/5 min (*t*(9) = 8.88; *p* < 0.001). In the presence of dl-TBOA, however, PEA (5 μM) still effectively inhibited the 4-aminopyridine-evoked glutamate release (5.8 ± 0.2 nmol/mg/5 min; F(2, 14) = 116.32, *p* < 0.05; Figure 2). By contrast, bafilomycin A1 (0.1 μM) reduced 4-aminopyridine (1 mM)-evoked glutamate release (*t*(9) = 11.98; *p* < 0.001), and significantly blocked the inhibitory effect of PEA (5 μM) on 4-aminopyridine-evoked glutamate release (Figure 2). In the five tested synaptosomal preparations, no statistical difference was observed between the release after bafilomycin A1 alone (2.9 ± 0.3 nmol/mg/5 min) and after the bafilomycin A1 and PEA treatment (2.8 ± 0.3 nmol/mg/5 min; *p* = 0.99). These data indicate the PEA-mediated modulation of the exocytotic pool of release, rather than an effect on the glutamate transporter responsible for cytosolic efflux.

### 2.3. Effect of PEA on Synaptosomal Cytosolic Ca^2+^ Levels and Membrane Potential

**Figure 3:**
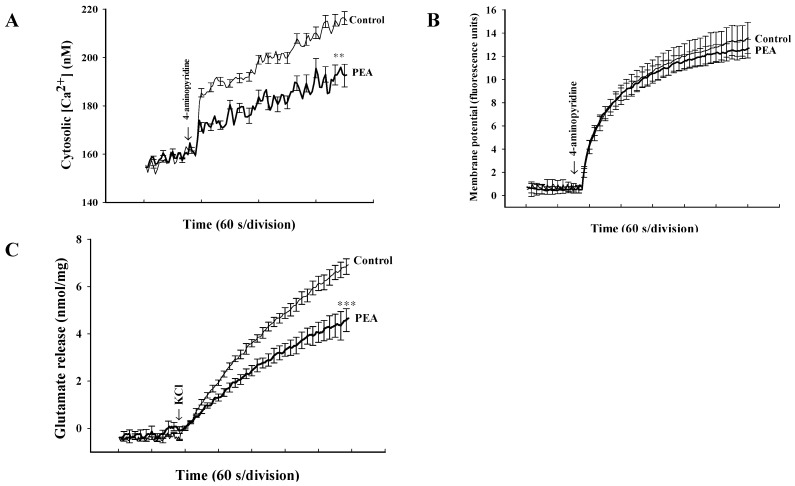
PEA reduces the 4-aminopyridine-induced increase in cytosolic Ca^2+^ concentration ([Ca^2+^]_C_) but fails to alter the synaptosomal membrane potential. The [Ca^2+^]_C_ (nM) (**A**) or synaptosomal membrane potential (**B**) was measured in the absence (control) and in the presence of 5 μM PEA, added 10 min before depolarization with 1 mM 4-aminopyridine. (**C**) Inhibition of KCl (15 mM)-evoked glutamate release by PEA. Results are mean ± SEM of 5 independent experiments. ** *p* < 0.01, *** *p* < 0.001 *versus* control group.

To further understand the mechanism of PEA-mediated inhibition of glutamate release, cytosolic Ca^2+^ levels were determined in synaptosomes preloaded with fura-2. In Figure 3a, stimulation of synaptosomes with 4-aminopyridine (1 mM) caused a rise in [Ca^2+^]_C_ from 158.3 ± 0.8 nM to a plateau level of 216.4 ± 2.7 nM. Preincubation of synaptosomes with PEA (5 μM) did not affect basal Ca^2+^ levels (159.8 ± 1.2 nM), but reduced the 4-aminopyridine-evoked rise in [Ca^2+^]_C_ (192.5 ± 4.7 nM; *t*(9) = 60.3; *p* < 0.01; Figure 3a). The observed inhibitory effect of PEA on the 4-AP-evoked increase in [Ca^2+^]_C_ might be attributed to the modulation of potassium channels and the consequently altered plasma membrane potential. To test this possibility, we used a membrane potential-sensitive dye, DiSC_3_(5) to determine the effect of PEA on the synaptosomal plasma membrane potential. Figure 3b shows that 4-aminopyridine (1 mM) caused an increase in DiSC_3_(5) fluorescence by 13.5 ± 1.2 fluorescence units/5 min. Preincubation of synaptosomes with PEA (5 μM) did not alter the resting membrane potential and produced no significant change in the 4-aminopyridine-mediated increase in DiSC_3_(5) fluorescence (12.8 ± 0.7 fluorescence units/5 min; *t*(9) = 0.56, *p* = 0.59). In addition, we observed the PEA-mediated inhibition of glutamate release using an alternative secretagogue, high external KCl concentrations. In Figure 3c, 15 mM KCl effected a release of 6.9 ± 0.2 nmol/mg/5 min, which decreased to 3.7 ± 0.1 nmol/mg/5 min in the presence of 5 μM PEA (*t*(9) = 10.68; *p* < 0.001). 

### 2.4. Effect of Ca^2+^ Channel Blockers and Intracellular Ca^2+^ Release Inhibitors on the Inhibition of Glutamate Release Mediated by PEA

**Figure 4:**
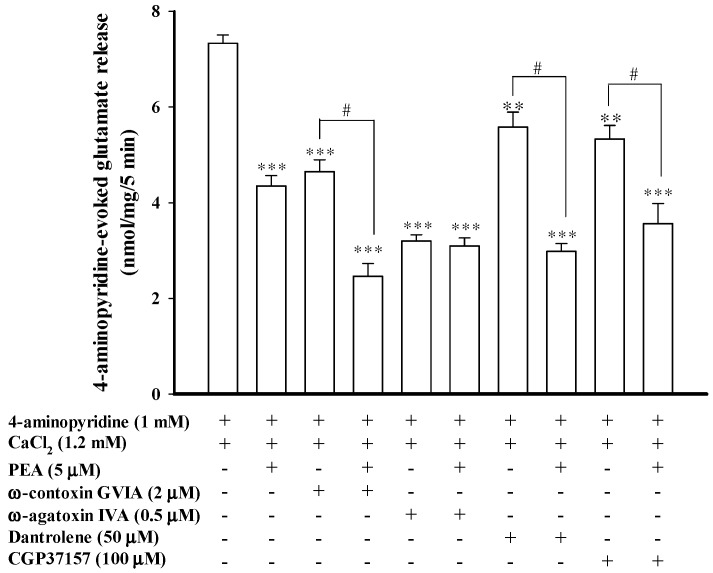
Blockade of Ca_v_2.1 (P/Q-type) channels prevents the inhibitory effect of PEA on 4-aminopyridine-evoked glutamate release. Bar graph showing glutamate release induced by 1 mM 4-aminopyridine in the absence (control) or presence of 5 μM PEA, 2 μM ω-conotoxin GVIA, 2 μM ω-conotoxin GVIA and 5 μM PEA, 500 nM ω-agatoxin IVA, 500 nM ω-agatoxin IVA and 5 μM PEA, 50 μM dantrolene, 50 μM dantrolene and 5 μM PEA, 100 μM CGP37157, or 100 μM CGP37157 and 5 μM PEA. PEA was added 10 min before depolarization, whereas the other drugs were added 30 min before depolarization. Results are mean ± SEM of 5–6 independent experiments. ** *p* < 0.01, *** *p* < 0.001 *versus* control group. ^# ^*p* < 0.05 *versus* the ω-conotoxin GVIA-, dantrolene- or CGP37157-treated group.

A reduction in the Ca^2+^-dependent release of glutamate could be explained by decreased entry of Ca^2+^ through Ca_v_2.2 (N-type) and Ca_v_2.1 (P/Q-type) channels, which can be selectively blocked by ω-conotoxin GVIA and by ω-agatoxin IVA, respectively [28,29,30,31]. To establish which of these Ca^2+^ channel activities was involved in the PEA-mediated inhibition of 4-aminopyridine-evoked glutamate release, we examined glutamate release in the presence of Ca^2+^ channel blockers. In Figure 4, the release of glutamate evoked by 1 mM 4-aminopyridine (7.2 ± 0.1 nmol/mg/5 min) decreased in the presence of 2 μM ω-conotoxin GVIA (4.6 ± 0.2 nmol/mg/5 min; *t*(11) = 9.79; *p* < 0.001) or 0.5 μM ω-agatoxin IVA (3.2 ± 0.1 nmol/mg/5 min; *t*(9) = 22.03; *p* < 0.001). In the presence of ω-conotoxin GVIA (2 μM), the application of PEA (5 μM) still effectively inhibited 4-aminopyridine-evoked glutamate release (2.5 ± 0.3 nmol/mg/5 min; F(2, 20) = 122.12, *p* < 0.05). By contrast, the inhibitory effect of PEA on 4-aminopyridine-evoked glutamate release was prevented in the presence of ω-agatoxin IVA. The release measured in the presence of both ω-agatoxin IVA and PEA (3.1 ± 0.2 nmol/mg/5 min) was similar to that obtained in the presence of ω-agatoxin IVA alone (3.2 ± 0.1 nmol/mg/5 min; *p* = 0.87; Figure 4). Therefore, a reduction in Ca^2+^ influx mediated by Ca_v_2.1 (P/Q-type) channels seems to be associated with the observed inhibition of glutamate release by PEA. 

We also tested the effect of dantrolene, an inhibitor of intracellular Ca^2+^ release from the endoplasmic reticulum, and 7-chloro-5-(2-chloropheny)-1,5-dihydro-4,1-benzothiazepin-2(3H)-one (CGP37157), a membrane-permeant blocker of mitochondrial Na^+^/Ca^2+^ exchange, on the PEA inhibition of glutamate release. Figure 4 shows that dantrolene (50 μM) reduced the 4-aminopyridine (1 mM)-evoked release (*t*(9) = 3.47; *p* < 0.01). In the presence of dantrolene, however, PEA (5 μM) still effectively inhibited 4-aminopyridine-evoked glutamate release. In the five tested synaptosomal preparations, a statistical difference was observed between the release after dantrolene alone (5.6 ± 0.3 nmol/mg/5 min) and after the dantrolene and PEA treatment (2.9 ± 0.2 nmol/mg/5 min) (F(2, 14) = 67.11; *p* < 0.05; Figure 4). Similarly to dantrolene, CGP37157 (100 μM) reduced the release of glutamate evoked by 1 mM 4-aminopyridine (*p* < 0.01), and it had no effect on the PEA-mediated inhibition of evoked glutamate release (Figure 4). These results suggested that a reduction of intracellular Ca^2+^ release appears not to mediate the inhibitory effect of PEA on 4-aminopyridine-evoked glutamate release.

**Figure 5:**
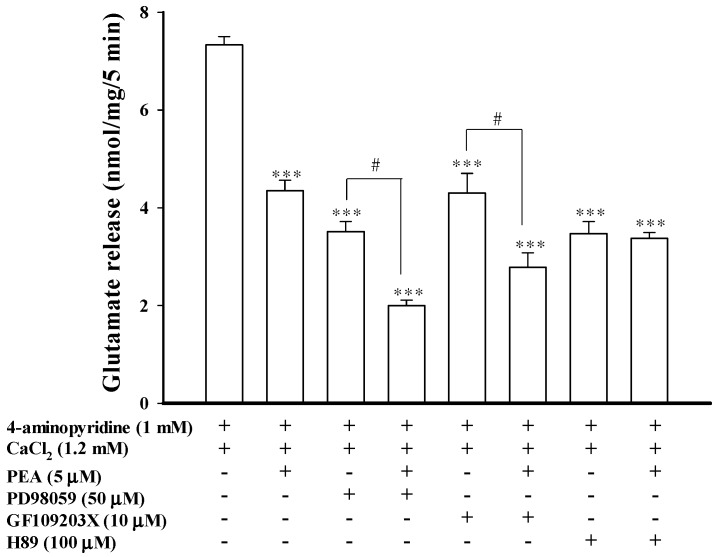
PEA-mediated inhibition of evoked glutamate release is prevented by the PKA inhibitor H89. Bar graph showing glutamate release induced by 1 mM 4-aminopyridine in the absence (control) or presence of 5 μM PEA, 50 μM PD98059, 50 μM PD98059 and 5 μM PEA; 10 μM GF109203X, 10 μM GF109203X and 5 μM PEA; 100 μM H89; or 100 μM H89 and 5 μM PEA. PEA was added 10 min before depolarization, whereas the other drugs were added 30 min before depolarization. Results are mean ± SEM of 5–7 independent experiments. *** *p* < 0.001 *versus* control group. ^#^ *p* < 0.05 *versus* the PD98059- or GF109203X-treated group.

### 2.5. Effect of MEK, PKC, and PKA Inhibitors on the Inhibition of Glutamate Release Mediated by PEA

To identify the intrasynaptosomal enzymatic pathways participating in the PEA inhibition of glutamate release, we tested several enzyme inhibitors: 2-(2-amino-3-methoxyphenyl)-4*H*-1-benzopyran-4-one (PD98059), a mitogen-activated/extracellular signal-regulated kinase (MEK) inhibitor; bisindolylmaleimide I (GF109203X), a protein kinase C (PKC) inhibitor and *N*-[2-(p-bromocinnamylamino)-ethyl]-5-isoquinolinesulfonamide dihydrochloride (H89), a protein kinase A (PKA) inhibitor. As illustrated in Figure 5, PD98059 (50 μM) reduced 4-aminopyridine (1 mM)-evoked glutamate release (7.2 ± 0.2 nmol/mg/5 min) to 3.4 ± 0.1 nmol/mg/5 min (*t*(13) = 14.59; *p* < 0.01). In synaptosomes pretreated with PD98059, PEA (5 μM) was still able to reduce 4-aminopyridine-evoked glutamate release (1.9 ± 0.1 nmol/mg/5 min) (Figure 5). A statistical difference was observed between the release after PD98059 alone and after the PD98059 and PEA treatment (F(2, 20) = 258.24; *p* < 0.05). Similarly, GF109203X (10 μM) reduced the release of glutamate evoked by 4-aminopyridine (1 mM) to 4.2 ± 0.4 nmol/mg/5 min (*t*(9) = 8.01; *p* < 0.001). In the presence of GF109203X and PEA, the inhibition of glutamate release following 4-aminopyridine-depolarization was significantly different from the effect of GF109203X alone (2.8 ± 0.3 nmol/mg/5 min; F(2, 14) = 66.73; *p* < 0.05) (Figure 5). By contrast, H89 (100 μM) reduced the 4-aminopyridine (1 mM)-evoked glutamate release (*t*(13) = 11.34; *p* < 0.001), and largely prevented the inhibition of glutamate release by PEA (5 μM). The release measured in the presence of H89 and PEA (3.4 ± 0.1 nmol/mg/5 min) was similar to that obtained in the presence of H89 alone (3.5 ± 0.2 nmol/mg/5 min; *p* = 0.94; Figure 5). These results indicated that PEA-inhibited glutamate release involves a PKA pathway.

### 2.6. Effect of the Cannabinoid CB1 Receptor Antagonist AM281, the TRPV1 Antagonist Capsazepine, or the PPARα Antagonist GW6471, on the Inhibition of Glutamate Release Mediated by PEA

**Figure 6:**
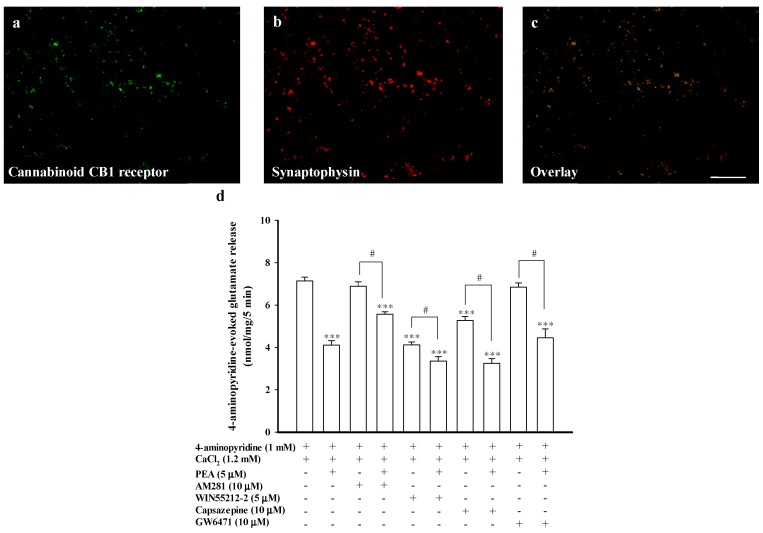
PEA-mediated inhibition of evoked glutamate release is partially blocked by the selective cannabinoid CB1 receptor antagonist AM281. Synaptosomes were fixed onto polylysine-coated coverslips and double stained for immunocytochemistry with antisera against cannabinoid CB1 receptors (red in (**a**)) and the vesicular marker synaptophysin (green in (**b**)). Merged panels are shown in (**c**) (orange). Scale bar, 10 μm; (**d**) Bar graph showing glutamate release induced by 1 mM 4-aminopyridine in the absence (control) or presence of 5 μM PEA, 10 μM AM281 (an antagonist of cannabinoid CB1 receptor), 10 μM AM281 and 5 μM PEA; 5 μM WIN55212-2 (an agonist of cannabinoid CB1 receptor), 5 μM WIN55212-2 and 5 μM PEA; 10 μM capsazepine (an antagonist of TRPV1); 10 μM capsazepine and 5 μM PEA; 10 μM GW6471 (an antagonist of PPARα); or 10 μM GW6471 and 5 μM PEA. Results are mean ± SEM of 5–7 independent experiments. *** *p* < 0.001 *versus* control group. ^# ^*p* < 0.05 *versus* the AM281-treated group.

Previous research has suggested that the central effects of PEA are mediated by an indirect activation of cannabinoid CB1 receptors [32,33]. The cannabinoid CB1 receptors are present at the presynaptic level, and their activation has been shown to inhibit Ca^2+^ influx and glutamate release [34,35]. As previously suggested [34], the cerebrocortical nerve terminal preparation from adult rats is enriched in this receptor, as witnessed by co-labeling with antisera against the presynaptic marker synaptophysin and the cannabinoid CB1 receptors expressed in the synaptosomes (Figure 6a–c). To determine whether the cannabinoid CB1 receptor was involved in the inhibition of glutamate release by PEA, we examined the effect of 1-(2,4-dichlorophenyl)-5-(4-iodophenyl)-4-methyl-*N*-4-morpholinyl-1*H*-pyrazole-3-carboxamide (AM281), an antagonist of the cannabinoid CB1 receptor, on the action of PEA. Figure 6d shows that AM281 (10 μM) had no effect on the 4-aminopyridine (1 mM)-evoked glutamate release (6.9 ± 0.2 nmol/mg/5 min; *t(*14) = −0.93; *p* = 0.37). In the presence of AM281, however, the inhibitory effect of PEA (5 μM) on 4-aminopyridine-evoked glutamate release was partially prevented (Figure 6d). On average, PEA resulted in an 18.8% ± 3.6% inhibition on 4-aminopyridine-evoked glutamate release after treatment with AM281 (5.6 ± 0.1 nmol/mg/5 min), which was less than that of the inhibition produced by PEA alone (42.3% ± 5.5%) (F(2, 20) = 22.67, *p* < 0.05). Additionally, 5 μM *R*-(+)-(2,3-dihydro-5-methyl-3-[(4-morpholiny)methyl]pyrrolo[1,2,3,-de]-1,4-benzoxazin-6-yl)(1-naphthalenyl)methanone monomethanesulfonate (WIN55212-2), a potent CB1 receptor agonist, inhibited 4-aminopyridine-evoked glutamate release (4.1 ± 0.1 nmol/mg/5 min; 41.4% ± 3.5% inhibition; *p <* 0.001), which was similar to the inhibition produced by PEA alone (42.3% ± 5.5%; *p* > 0.05; Figure 6d). In the presence of WIN55212-2, PEA (5 μM) continued to significantly reduce the release of glutamate evoked by 4-aminopyridine (3.3 ± 0.2 nmol/mg/5 min; 17.1% ± 4.3%; F(2, 11) = 80.53, *p* < 0.05 ), but this inhibition was significantly different from the inhibition produced by PEA alone (42.3% ± 5.5%; *p* < 0.05; Figure 6d). On the other hand, transient receptor potential vanilloid type-1 (TRPV1) and peroxisomal proliferator activated receptor α (PPARα) have been reported to mediate the action of PEA [1,3]. Therefore, we also determined whether directly antagonizing TRPV1 or PPARα can prevent the inhibitory effect of PEA on glutamate release. In Figure 6d, capsazepine (10 μM), a TRPV1 selective antagonist, reduced 4-aminopyridine-evoked glutamate release (5.3 ± 0.2 nmol/mg/5 min; *p <* 0.001). In the presence of capsazepine, PEA (5 μM) still effectively inhibited 4-aminopyridine-evoked glutamate release (3.3 ± 0.2 nmol/mg/5 min). A statistical difference was observed between the release after capsazepine alone and after the capsazepine and PEA treatment (F(2, 13) = 79.23; *p <* 0.005; Figure 6d). GW6471 (10 μM), a PPARα selective antagonist, exerted no effect on either control 4-aminopyridine-evoked glutamate release (*p* = 0.924) or inhibition of glutamate release by PEA. The release measured in the presence of GW6471 and PEA (4.5 ± 0.4 nmol/mg/5 min) was significantly different from that obtained in the presence of GW6471 alone (6.9 ± 0.2 nmol/mg/5 min; F(2, 13) = 24.59; *p* < 0.05; Figure 6d). These results indicated that PEA inhibits glutamate release at least in part through cannabinoid CB1 receptor activation.

## 3. Discussion

Studies have suggested that several neuroprotectants may work by stabilizing glutamate release when its synaptic level becomes too high, a feature that is now considered to be part of the pathophysiology of numerous neurological disorders [20]. PEA, a member of the fatty-acid ethanolamide family, is an important neuroprotective mediator in the brain [4]; however, the cellular mechanism responsible for its neuroprotective effect is still debated. Using a biochemical study in cerebrocortical nerve terminals, we found that PEA inhibits the Ca^2+^-dependent exocytosis of glutamate evoked by 4-aminopyridine with an IC_50_ value in the micromolar range. Based on our research, no other studies have investigated the effect of PEA on endogenous glutamate release. This is the first report to document the mechanism by which PEA inhibits glutamate release at the presynaptic level.

### Mechanism of Action of PEA in the Inhibition of Glutamate Release

In principle, glutamate release can be modulated at multiple loci in the stimulus-exocytosis cascade, including ion-channel modulating nerve terminal excitability, voltage-dependent Ca^2+^ channels, and the release process itself [26,36]. The inhibition of Na^+^ channels and the activation of K^+^ channels are recognized to result in presynaptic inhibition resulting from the stabilization of membrane excitability. This causes a subsequent decrease in voltage-dependent Ca^2+^ entry and a consequent reduction of neurotransmitter release [37,38]. In the present study, our data suggest that the observed inhibition of 4-aminopyridine-evoked glutamate release by PEA is not due to a reduction of nerve terminal excitability caused by ion channel (e.g., the Na^+^ or K^+^ channels) modulation. First, PEA inhibited the release of glutamate evoked by 4-aminopyridine and KCl. This indicates that Na^+^ channels are not involved in the effect of PEA on glutamate release, because 4-aminopyridine-evoked glutamate release involves the action of Na^+^ and Ca^2+^ channels and 15 mM external KCl-evoked release involves only Ca^2+^ channels [26,39]. Second, no effect of PEA on the synaptosomal membrane potential, measured using a membrane potential dye DiSC_3_(5), was observed either under resting conditions or on depolarizing with 4-aminopyridine. This indicates that the observed inhibition of glutamate release by PEA is not due to an augmentation of K^+^ conductance.

By using Fura-2, we demonstrated that a decrease in intraterminal Ca^2+^ levels is necessary for the observed PEA-mediated decrease of glutamate release. Our finding is consistent with those of previous studies that have demonstrated that PEA reduces the depolarization- or bradykinin-induced Ca^2+^ transient increases in cultured dorsal root ganglion neurons and differentiated F11 cells [40,41]. However, Ambrosino *et al.* [40] found that PEA increases intracellular Ca^2+^ concentrations in differentiated F11 cells, suggesting that different mechanisms of action might be involved. In addition, the observed inhibitory effect of PEA on 4-aminopyridine-evoked glutamate release was found to be prevented by chelating extracellular Ca^2+^ ions and the Ca_v_2.1 (P/Q-type) channel blocker ω-agatoxin IVA. However, ω-conotoxin GVIA, a Ca_v_2.2 (N-type) channel blocker, dantrolene, an inhibitor of intracellular Ca^2+^ release from the endoplasmic reticulum ryanodine receptors, and CGP37157, a mitochondrial Na^+^/Ca^2+^ exchange blocker, had no effect on the action of PEA. Therefore, these results demonstrate that a reduction in Ca^2+^ influx mediated by Ca_v_2.1 (P/Q-type) channels is associated with the inhibition of glutamate release by PEA, whereas a decrease of intracellular store Ca^2+^ release seems not to be involved.

How PEA affects the Ca_v_2.1 (P/Q-type) channels remains unclear. The effect of PEA on presynaptic Ca_v_2.1 (P/Q-type) channels might be attributable to either the direct effect on Ca^2+^ channel function or secondary effects caused by, for example, the modulation of protein kinase, and the consequently altered voltage-dependent Ca^2+^ channel phosphorylation. These possibilities need to be elucidated by additional studies. However, in this study, a role of PKA in the PEA-mediated inhibition of glutamate release is suggested. This stems from the observation that the PKA inhibitor H89 prevented the inhibitory effect of PEA on glutamate release. Moreover, the inhibitory effect of PEA on 4-aminopyridine-evoked glutamate release was not affected by the MEK inhibitor PD98059 and the PKC inhibitor GF109203X, which demonstrated some specificity for PEA action on the PKA pathway. In nerve terminals, PKA is known to phosphorylate voltage-dependent Ca^2+^ channels and several synaptic proteins, subsequently increasing glutamate release [42,43]. Therefore, a reduction of PKA-mediated phosphorylation of synaptosomal Ca_v_2.1 (P/Q-type) channels should be considered when determining the possible mechanism of PEA-mediated presynaptic inhibition.

However, several actions of PEA such as anti-nociceptive and anti-epileptic effects are reported to be associated with cannabinoid CB1 receptor activation [32,33]. Other studies have proposed the cannabinoid CB1 receptor-independent pathway by PEA [1,7,44]. The cannabinoid CB1 receptor is present in numerous regions of the brain including the cerebral cortex and is found both pre- and post-synaptically. At the presynaptic level, activation of cannabinoid CB1 receptors has been reported to inhibit Ca^2+^ influx and glutamate release [34,35]. In the present study, we observed that cannabinoid CB1 receptors were coexpressed with the presynaptic marker synaptophysin, confirming that cannabinoid CB1 receptors are present in the cerebrocortical nerve terminals, which is consistent with the finding of a previous study [34]. However, the selective cannabinoid CB1 receptor antagonist AM281 could not fully block the action of PEA on 4-aminopyridine-evoked glutamate release (about 19% of inhibition remained). Moreover, activating the cannabinoid CB1 receptors also partially prevented the PEA-mediated inhibition of glutamate release. Therefore, we cannot rule out the possibility that receptors other than cannabinoid CB1 receptors or other non-receptor pathways might be involved in the action of PEA on glutamate release. For example, TRPV1, PPARα, and orphan G protein-coupled receptor 55 are reported to be involved in the action of PEA [1,3]. In the present study, TRPV1 and PPARα could be excluded. This is because of the inhibitory effects of PEA on the 4-aminopyridine-evoked glutamate release were not affected by the TRPV1 antagonist capsazepine or the PPARα antagonist GW6471. Although PEA does not bind to cannabinoid CB1 receptors, and some inconsistency exists among different studies [1,45], our results indicate that the cannabinoid CB1 receptors are present in cerebrocortical nerve terminals, and their activity is partially involved in the PEA-inhibited glutamate release.

## 4. Experimental Section

### 4.1. Chemicals

PEA, AM281, dl-TBOA, bafilomycin A1, dantrolene, CGP37157, GF109203X, PD98059, WIN55212-2, capsazepine, and GW6471 were purchased from Tocris Cookson (Bristol, UK). 3',3',3'-dipropylthiadicarbocyanine iodide (DiSC_3_(5)), and fura-2-acetoxymethyl ester (Fura-2-AM) were purchased from Invitrogen (Carlsbad, CA, USA). 4-aminopyridine, ω-conotoxin GVIA, ω-agatoxin IVA, H89, ethylene glycol bis (β-aminoethyl ether)-*N*,*N*,*N'*,*N'*-tetraacetic acid (EGTA), and all other reagents were purchased from Sigma-Aldrich Co. (St. Louis, MO, USA).

### 4.2. Animals

Adult male Sprague-Dawley rats (150–200 g) were purchased from BioLASCO (Taiwan Co., Ltd., Taipei, Taiwan). Animals were housed at constant temperature (22 ± 1 °C) and relative humidity (50%) under a regular 12 h light-dark cycle (lights off at 7 pm). Food and water were freely available. The animals were sacrificed by decapitation and the cerebral cortex rapidly removed at 4 °C. The experimental procedures were approved by the Institutional Animal Care and Use Committee at the Far-Eastern Memorial Hospital, in accordance with the National Institutes of Health Guide for the Care and Use of Laboratory Animals. All efforts were made to minimize animal suffering and to use a minimum number of animals necessary to produce reliable results.

### 4.3. Preparation of Synaptosomes

Synaptosomes were prepared on Percoll gradients as previously described [25,46]. The final synaptosomal fraction was resuspended in a HEPES-buffered medium (HBM) consisting of 140 mM NaCl, 5 mM KCl, 5 mM NaHCO_3_, 1 mM MgCl_2_·6H_2_O, 1.2 mM Na_2_HPO_4_, 10 mM glucose, and 10 mM HEPES at pH 7.4. Protein concentration was determined using a Bradford assay. Synaptosomes were centrifuged in a final wash to obtain synaptosomal pellets containing 0.5 mg of protein. The synaptosomal pellets were stored on ice and used within 4–6 h.

### 4.4. Glutamate Release Assay

Glutamate release was measured by online fluorometry [25]. Synaptosomal pellets were resuspended at a protein concentration of 0.5 mg/mL in HBM containing 16 μM bovine serum albumin (BSA) and incubated in a stirred and thermostated cuvette maintained at 37 °C in a PerkinElmer LS-55 spectrofluorometer (PerkinElmer Life and Analytical Sciences, Waltham, MA, USA). NADP^+^ (2 mM), glutamate dehydrogenase (50 units/mL), and CaCl_2_ (1.2 mM) were added after 3 min. After a further 10 min of incubation, 4-aminopyridine (1 mM) or KCl (15 mM) was added to stimulate glutamate release. The oxidative deamination of the released glutamate, leading to the reduction of NADP^+^, was monitored by measuring NADPH fluorescence at excitation and emission wavelengths of 340 and 460 nm, respectively. Data were accumulated at 2 s intervals. A standard of exogenous glutamate (5 nmol) was added at the end of each experiment, and the fluorescence response was used to calculate the released glutamate as nanomoles glutamate per milligram synaptosomal protein (nmol/mg). Release traces are shifted vertically to align the point of depolarization as zero release. Release values quoted in the text and depicted in bar graphs represent the levels of glutamate cumulatively released after 5 min of depolarization, and are expressed as nmol/mg/5 min. Cumulative data were analyzed using Lotus 1-2-3 (IBM, White Plains, NY, USA).

### 4.5. Cytosolic Ca^2+^ Concentration ([Ca^2+^]_C_)

Synaptosomes (0.5 mg/mL) were preincubated in HBM containing 5 μM fura-2-AM, 0.1 mM CaCl_2_, and 16 μM BSA for 30 min at 37 °C in a stirred test tube. After fura-2 loading, the synaptosomes were centrifuged in a microcentrifuge for 1 min at 10,000× *g*. The synaptosomal pellets were resuspended in HBM containing BSA, and placed in a PerkinElmer LS-55 spectrofluorometer at 37 °C with stirring in the presence of 1.2 mM CaCl_2_. The synaptosomes were incubated for 10 min in the presence of PEA (5 μM) prior to being depolarized with 4-aminopyridine (1 mM). Fura-2-Ca fluorescence was determined at excitation wavelengths of 340 and 380 nm (emission wavelength, 505 nm), and data were accumulated at 2 s intervals. [Ca^2+^]_C_ (nM) was calculated by using calibration procedures [47] and equations described previously [48]. Cumulative data were analyzed using Lotus 1-2-3.

### 4.6. Synaptosomal Plasma Membrane Potential

The synaptosomal membrane potential can be determined by positively charged membrane potential-sensitive carbocyaninedyes such as DiSC_3_(5). The dye becomes incorporated into the synaptosomal plasma membrane lipid bilayer. Upon depolarization with 4-aminopyridine, the release of dye from the membrane bilayer is indicated as an increase in fluorescence [49]. Synaptosomes were resuspended in HBM and incubated in a stirred and thermostated cuvette at 37 °C in a PerkinElmer LS-55 spectrofluorometer. After 3 min incubation, 5 μM DiSC_3_(5) was added and allowed to equilibrate before the addition of 1.2 mM CaCl_2_ or PEA (5 μM). 4-aminopyridine was added to depolarize the synaptosomes at 10 min, and DiSC_3_(5) fluorescence was measured at excitation and emission wavelengths of 646 and 674 nm, respectively. Data were accumulated at 2 s intervals. Cumulative data were analyzed using Lotus 1-2-3, and results are expressed in fluorescence units.

### 4.7. Immunocytochemistry

The synaptosomes were allowed to attach to coverslips (diameter 20 mm) precoated with poly-l-lysine for 40 min at 4 °C before being fixed with 4% paraformaldehyde in 0.1 M phosphate buffer (pH 7.4) for 30 min. After rinsing with phosphate buffer three times, the synaptosomes were incubated in blocking buffer containing 3% normal goat serum and 0.2% Triton X-100 for 60 min. They were then incubated with a mixture of primary mouse monoclonal antibodies against synaptophysin (1:200; Abcam, Cambridge, UK) and rabbit monoclonal antibodies against cannabinoid CB_1_ receptor (1:100; Abcam) for 90 min at room temperature. After rinsing with blocking buffer, the synaptosomes were incubated with a mixture of goat anti-mouse DyLight 549- and goat anti-rabbit fluoresce in isothiocyanate (FITC)-conjugated secondary antibodies (1:200; Jackson ImmunoResearch Inc., West Grove, PA, USA) for 1 h at room temperature. The synaptosomes were then washed three times with phosphate buffer and 0.1 M carbonate buffer (pH 9.2), and coverslipped with fluorescence mounting medium (DAKO North America, Inc., Carpinteria, CA, USA). Double immunofluorescence images were observed at a magnification of 400×, using upright fluorescence microscopy (LeicaDM2000 LED, Wetzlar, Germany), and images were captured using a CCD camera (SPOT RT3, Diagnostic Instruments, Sterling Heights, MI, USA).

### 4.8. Statistical Analysis

Data were expressed as mean ± SEM. The data reported were analyzed by using the unpaired Student’s *t* test or by using one-way ANOVA accompanied by Tukey’s test for multiple comparisons. Analysis was completed via software SPSS (17.0; SPSS Inc., Chicago, IL, USA). *p* < 0.05 was considered to represent a significant difference.

## 5. Conclusions

Our data show that PEA exerts an inhibitory effect on the evoked glutamate release from cerebrocortical nerve terminals by a mechanism that involves the suppressing of Ca_v_2.1 (P/Q-type) channels and PKA activity. Furthermore, this release inhibition likely depends, at least in part, on the activation of cannabinoid CB1 receptors. Because the excitotoxicity caused by excessive glutamate release is a critical element in the pathogenesis of acute and chronic brain disorders [15], the ability of PEA to depress glutamate release may be one of the mechanisms underlying neuroprotection. This investigation extends our knowledge on the mode of PEA action in the brain and provides a rationale for using PEA to treat brain disorders.
